# Relationships between Weight, Adiposity, Functional Status, and Left Ventricle Characteristics in Overweight and Obese Patients with Heart Failure

**DOI:** 10.15436/2376-0494.17.1108

**Published:** 2017-02-20

**Authors:** Marjan Motie, Lorraine S. Evangelista, Dawn Lombardo, Tamara B. Horwich, Michele Hamilton, Gregg C. Fonarow

**Affiliations:** 1 Program in Nursing Science, University of California Irvine, Irvine, California, USA; 2 Department of Medicine, University of California Irvine Medical Center, Irvine, California, USA; 3 University of California at Los Angeles, David Geffen School of Medicine, Los Angeles, California, USA; 4 Department of Medicine/Cardiology UCLA and Director of the Heart Failure Program, Cedars-Sinai Heart Institute, Los Angeles, California, USA; 5 Director, UCLA Cardiomyopathy Center and Professor of Cardiology, David Geffen School of Medicine, Los Angeles, California, USA

**Keywords:** Heart failure, Functional status, Left ventricular characteristics, Obesity

## Abstract

**Aims:**

This study was conducted to examine the relationship between adiposity and functional status (i.e. peak oxygen consumption [VO_2_max]), and left ventricular (LV) structural characteristics (i.e., LV ejection fraction [LVEF], LV end diastolic dimension [LVEDD], LV posterior wall thickness [LVPWT]) in heart failure (HF) patients with diabetes mellitus (DM), and/or metabolic syndrome (MS). We hypothesize that excess weight and body fat are significantly related to cardiac functional status.

**Methods and Results:**

Ninety four patients’ clinical characteristics were analyzed at baseline to examine the relationships of interest. Results show that weight was correlated with fat and lean mass and LVEF (all p’s < 0.050). Novel findings from our data showed that weight, fat mass, and percent fat were inversely related to VO_2_max; weight, fat mass and lean mass were positively related with LVPWT. In a multivariate analysis, body mass index and fat mass accounted for 28.8% of the variance in VO_2_max, showing significantly higher predictive value than other covariates (P = 0.002).

**Conclusions:**

Our findings show a possible relationship between body fat on functional status in this patient cohort and challenges existing research that supports that higher weight and increased fat are good in the setting of chronic HF (i.e. obesity paradox). Strategies to optimize weight and reduce adiposity warrants further investigation in this subgroup of patients.

## Introduction

Chronic heart failure (HF) is a healthcare epidemic characterized by progressive decline of cardiac performance and continues to be a public health problem affecting over 5 million Americans^[1]^. Chronic HF is associated with significant comorbidities and frequent decompensation, resulting in recurrent hospitalizations^[2,3]^. Being overweight or obese are well established comorbidities and/or risk factors that lead to health consequences including coronary heart disease, hypertension, diabetes mellitus (DM), dyslipidemia, sleep apnea, and certain cancers^[4]^.

Obesity has been reported as affecting the cardiovascular system directly in many ways, in addition to the indirect consequences caused by the deleterious effects of the known comorbidities and their associated risk factors^[5]^. Many human and animal studies have led to the understanding that obesity affects the structure and function of the heart mainly by causing increased blood volume, elevated cardiac output, left ventricular (LV) hypertrophy, and LV diastolic dysfunction all of which also play a role in causing HF^[6]^.

Recent literature has described the relationship between obesity and overweight with several obesity related complications (e.g., cardiac functional status, LV characteristics)^[7]^; however, these relationships are even less well defined in patients with HF and multiple co-morbidities (e.g., obesity, DM, metabolic syndrome [MS]). Prior research has not been able to distinctly identify the effects of obesity from the effects of the comorbidities which on their own cause LV dysfunction. Furthermore, despite the known deleterious effects of excess body weight as an independent risk factor for cardiovascular disease (CVD), there are multiple studies showing improved survival in obese patients with known CVD (concept known as obesity paradox)^[8,9–13]^. The specific aims of this investigation are to: 1) examine the association between overall weight, body composition (i.e., fat mass, lean mass, percent body fat), and structural and functional cardiac status in this sample of HF patients; and 2) identify independent predictors of functional status to gain some insight into the possible nature of the deleterious vs. beneficial effects of obesity in HF.

## Methods

The enrollment criteria and study design have been described elsewhere^[14]^. Briefly, 94 participants were recruited and provided informed consent to participate in a randomized controlled trial for overweight and obese patients with NYHA Functional Class II-IV HF, DM, and/or MS, irrespective of LVEF (Table 1). The University of California Los Angeles and University of California Irvine Institutional Human Subjects Review Committees approved the study. The investigation conformed with the principles outlined in the Declaration of Helsinki.15 Participants who met the inclusion/exclusion criteria received a 3-month behavioral weight management intervention based on one of two different macronutrient content energy-restricted meal plans (at 1200 or 1500 kcal/day) which would provide a calorie deficit aimed at 500kcal or more. For the purpose of this descriptive study, weight, adiposity (i.e., fat mass, lean mass, percent body fat), peak oxygen consumption [VO_2_max]) -as measured by cardiopulmonary exercise test (CPX) - and LV structural characteristics (i.e. LV ejection fraction [LVEF], LV end diastolic dimension [LVEDD], LV posterior wall thickness [LVPWT] –as measured by echocardiogram- were described for all participants at baseline.

Data was analyzed using SPSS version 19.0 for Windows. Sociodemographic and clinical variables were computed using descriptive statistics (e.g., means and standard deviations for continuous variables and 2 tests for categorical variables). Relationships between variables of interest (weight, body mass index [BMI], fat mass, lean mass, fat %, VO_2_max, LVEF, LVEDD, and LVPWT) were analyzed using Pearson Moment correlations. Multivariate analyses were performed to test the independent association between cardiac function (as measured by VO_2_max) and adiposity and BMI (the dependent variables) with age, gender, New York Heart Association (NYHA) class, history of diabetes or hypertension as covariates in one model and the same covariates plus LVEF and LVPWT in another model.

## Results

### Participant characteristics

Table 1 shows the sociodemographic and clinical characteristics of the study sample. Participants ranged in age from 27 to 81 and were on the average moderately obese (BMI, 37.08 ± 6.18 kg/m^2^, range 27 to 61). Participants’ baseline clinical characteristics and cardiac structural and functional measurements are also shown in Table 1.

### Association between weight and body composition and cardiac function

The correlation matrix for key variables is illustrated in Table 2. As shown, weight and BMI were correlated with fat and lean mass and LVEF (all p’s < 0.050). Weights, BMI, fat mass and percent fat were inversely related to VO_2_ max; while weight and lean mass were positively related with LVPWT.

### Subgroup Analysis

The multivariate analyses showed that the variance in the outcome, VO_2_ max, explained by all the predictors in a model with age, gender, NYHA class, history of diabetes or hypertension is not different from a model with any of the predictors (F (5,39) = 1.97, P = 0.105) as shown in Table 3, model 1. Table 3, model 2 illustrates that sequentially adding LVPWT and LVEF to the list of predictors shows no further contribution to explaining the variance in VO_2_ max (F (2,37) = 1.175, P = 0.320). In model 3, the subsequent addition of BMI and fat as predictors improves the previous models significantly (P = 0.007). Overall, fat mass along with BMI accounted for 28.8% of the variance in VO_2_ max while the cardiac structural measures (LVEF and LVPWT) had much lower predictive correlations (10.7%).

## Discussion

To our knowledge, this is the first study to explore the relationship between adiposity and cardiac structure and function in overweight and obese patients with HF who also have DM and/or MS. Although the higher incidence of CVD in obese individuals has been extensively reported as being linked to known risk factors such as dyslipidemia, hypertension, glucose intolerance, inflammatory markers, and obstructive sleep apnea,^[5;6;16]^ the relationship between obesity and the structural and functional characteristics of the heart itself has not been fully explored in this population.

Several studies have recently described the possible effects of obesity on the left ventricle^[16–19]^. However, the pathogenesis of LV dysfunction in obesity is not completely understood but has been linked to an increase in plasma volume and long term elevation in cardiac output (as characterized by dilated, hypertrophied ventricles and increased stroke volume), both resulting from the amplified metabolic demands of both excessive fatty tissue and lean body mass.^[5,6]^ Obesity was reported as being associated with concentric LV remodeling with no accompanying change in ejection fraction in a large population based study^[18]^. In obese adolescent girls, the onset of cardiac dysfunction and the extent to which LV size, stroke volume and cardiac output increased were reported to be related to the severity of obesity^[20]^. In HF patients, Lavie and colleagues^[21]^ concluded that there were no significant differences in LV ejection phases between lean and obese patients (independent of HF etiology).

Considering the known adverse effects of obesity on LV structure and function, the findings of a recent report that demonstrated a positive correlation between BMI and worse diastolic function in a community based elderly cohort comes as no surprise^[17]^. This relationship was discussed as the possible reason for putting overweight and obese persons at increased risk for new onset HF since LV dysfunction is recognized as a possible pathophysiological link in the etiology of obesity induced HF. On the other hand, many studies have reported that once HF is established, obese individuals have a better overall clinical prognosis and reduced risk of mortality (referred to as the obesity paradox in HF)^[10,12,13,22,23]^. Our findings show that BMI is positively correlated with LVEF. These results appear to be in agreement with the studies describing an apparent obesity paradox in terms of the overall better LV diastolic function in more obese HF patients. Our results further showed a positive correlation between LVEF and LVEDD (as expected) but no correlation between the latter and fat or lean mass or BMI. Although the LV dilation is not affected by increased weight, the LV weight (measured by LVPWT) was significantly correlated with weight and lean mass and not fat mass. This parameter is known to measure the muscle mass surrounding the heart such that lower lean mass would be expected to contribute to lower heart muscle mass. In the study of obese subjects done at necropsy,^[22,24]^ an increase in heart weight, wall thickness and LV hypertrophy were observed; however, while the mechanism of LV hypertrophy was once attributed to excess body fat, recent data has attributed fat-free mass as a stronger predictor of LV mass and LV hypertrophy^[22,25]^.

Despite the favorable change in cardiac diastolic function with excess body weight, the significant negative relationship between fat mass (and BMI) and VO_2_ max (the gold standard in the assessment of clinical status in HF) is a novel finding that supports our hypothesis that excess fat may not be protective in HF patients^[26]^. Furthermore, the deleterious association of excess weight on VO_2_ max was not significantly correlated with any of the cardiac structural and functional parameters.

Previous studies of CPX in HF have only identified a link to the etiology of HF where prognosis was improved in ischemic obese or overweight HF patients whereas non-ischemic patients^[11,27]^ had similar outcomes as normal weight patients. It has also been reported that the cardiorespiratory fitness (FIT) of HF patients is an important factor in the relationship between obesity and prognosis; those with high FIT (peak VO_2_ > 14 mL O_2_ · kg^−1^ · min^−1^), demonstrated good prognosis with no evidence of an obesity paradox, but also no evidence that obesity was associated with worse outcomes^[9,21]^. In another report, researchers found that particularly in women and obese patients, who generally have a higher body fat content, peakVO_2_ best estimates exercise capacity (as a prognostic factor in survival) when it is computed by dividing by lean mass (rather than total weight)^[28]^. This was expressed as being related to the importance of skeletal muscles in the pathophysiology of HF. Our findings from a multivariate analysis show that the difference in VO_2_max among overweight and obese HF patients is best explained by fat mass (and BMI) as the dependent variable while keeping other risk factors constant (gender, age, NYHA class, history of hypertension and diabetes, LVEF, and LVPWT). To the best of our knowledge, this is the first report of the relationship of VO_2_max and body composition in this population suggesting that intentional weight loss may potentially result in better outcomes particularly for those with lower VO_2_max.

The present report shows a trend toward worsening cardiac function with increasing weight in HF patients with DM or MS. We did not have a concurrent control group of overweight or obese patients with no HF etiology. Also, there were very few patients with more advanced HF (NYHA class 4) to delineate the associations of worsening disease versus associations of increasing weight. Finally, another limitation of the current study was the small sample size.

The results of this study provide evidence that greater fat mass in obese patients with HF along with DM, and/or MS is an independent predictor of worse functional capacity of the heart. Future studies are warranted to examine the effect of weight management interventions (and differentiating between different approaches to fat loss) on the changes associated with cardiac status over short and long term duration in this highly vulnerable population.

## Figures and Tables

**Table 1:** Sociodemographic and Clinical Characteristics (N = 94)

	Baseline All Subjects
**Age, years (Mean ± SD)**	58.79 ± 9.95
**Male (%)**	69.6%
**White (%)**	65.2%
**History of Diabetes**	26.6%
**History of Hypertension**	43.6%
**History of Smoking**	56.2%
**History of Statin use**	68.7%
**NYHA class, N (%)**	
**Class 2**	81.1%
**Class 3**	18.9%
**Weight (lbs)**	239.63 ± 46.78
**Body Mass Index (BMI)**	37.08 ± 6.18
**Percent Fat**	38.06 ± 7.44
**VO_2_max (ml/kg/min)**	12.31 ± 3.79
**LVEF (%)**	39.06 ± 13.65
**LVDD (mm)**	58.24 ± 10.58
**LVPWT (mm)**	10.82 ± 2.21

VO_2_max – peak oxygen consumption; LVEF – left ventricular ejection fraction; LVEDD – left ventricular end diastolic dimension; LVPTW – left ventricular posterior wall thickness

**Table 2:** Correlational Matrix of Key Variables of Interest at Baseline (N = 94)

Variable	1	2	3	4	5	6	7	8	9
1. BMI	1.000								
2. Body weight (lbs.)	0.816†	1.000							
3. Fat mass (g)	0.859†	0.702†	1.000						
4. Lean mass (g)	0.236*	0 .712†	0.038	1.000					
5. Total % fat	0.561†	0 .152	0 .787†	−0.565†	1.000				
6. VO_2_max	−0.365†	−0.221*	−0.419†	0.177	−0.455†	1.000			
7. LVEF (%)	0.277*	0.235*	0.209	0.023	0.123	0.035	1.000		
8. LVEDD	−0.025	0.051	−0.050	0.033	−0.048	−0.153	−0.622†	1.000	
9. LVPWT	0.242	0 .460†	0 .108	0 .569†	−0.211	0.021	−0.022	−0.010	1.000

* P <.05

† P < .001

**Table 3:** Multivariate Analysis of Variables

			Change Statistics		
	Adjusted R- Squared	Std Error of the Estimate	R square Change	F Change	Significant F Change
Model 1	0.099	3.59	0.202	1.97	0.105
Model 2	0.107	3.57	0.048	1.18	0.320
Model 3	0.288	3.19	0.185	5.71	0.007

Predictors: Model 1: Age, gender, New York Heart Association Class (NYHA) , history of hypertension and diabetes; Model 2: Age, gender, NYHA Class, history of hypertension and diabetes, LVEF and LVPWT; Model 3: Age, gender, NYHA Class, history of hypertension and diabetes, LVEF, LVPWT, FAT (gram) and BMI

Testing: VO_2_ max
